# Enhanced Recovery After Bariatric Surgery (ERABS): Clinical Outcomes from a Tertiary Referral Bariatric Centre

**DOI:** 10.1007/s11695-013-1151-4

**Published:** 2013-12-20

**Authors:** Sherif Awad, Sharon Carter, Sanjay Purkayastha, Sherif Hakky, Krishna Moorthy, Jonathan Cousins, Ahmed R. Ahmed

**Affiliations:** 1 Imperial Weight Centre, St. Mary’s Hospital, Imperial College Healthcare NHS Trust, London, UK; 2 Division of Gastrointestinal Surgery, Nottingham Digestive Diseases Centre NIHR Biomedical Research Unit, Nottingham University Hospitals NHS Trust, E-floor, West Block, Derby Road, Nottingham, NG7 2UH UK

**Keywords:** Morbid obesity, Gastric bypass, Sleeve gastrectomy, Gastric band, Laparoscopic, Enhanced recovery, Fast track, Length of stay, Complications, Bariatric surgery, Co-morbidities

## Abstract

There is paucity of data on Enhanced Recovery After Bariatric Surgery (ERABS) protocols. This feasibility study reports outcomes of this protocol utilized within a tertiary-referral bariatric centre. Data on consecutive primary procedures (laparoscopic gastric bypasses, sleeve gastrectomies and gastric bands) performed over 9 months within an ERABS protocol were prospectively recorded. Interventions utilized included shortened preoperative fasts, intra-operative humidification, early mobilization and feeding, avoidance of fluid overload, incentive spirometry, use of prokinetics and laxatives. Data collected included demographics, co-morbidities, morbidity, mortality, length of stay (LOS) and re-admissions. A total of 226 procedures (age [mean ± SD], 45 ± 11 years, median [interquartile range] BMI 44.9 [41.0–49.0] kg/m^2^) were undertaken: 150 (66 %) bypasses, 47 (21 %) sleeves and 29 (13 %) bands. Hypertension, diabetes mellitus, sleep apnea and limited mobility were present in 40 %, 34 %, 24 % and 9 % of patients, respectively. No anastomotic or staple line leaks/bleeds were encountered. Ten (4.4 %) patients developed postoperative morbidity (mainly respiratory complications). One death occurred from massive pulmonary embolus in a high-risk patient (despite insertion of preoperative-IVC filter). Respective mean ± SD LOS for bypasses, sleeves and bands were 1.88 ± 1.12, 2.30 ± 1.69 and 0.69 ± 0.81 days. Successful discharge on the first postoperative day was achieved in 37 % and 28 % of bypasses and sleeves, respectively. Day-case gastric bands were performed in 48 %. Thirty-day hospital re-admission occurred in six (2.7 %) patients. Applying an ERABS protocol was feasible, safe, associated with low morbidity, acceptable LOS and low 30-day re-admission rates. The presence of multiple medical co-morbidities should not preclude use of an ERABS protocol within bariatric patients.

## Introduction

Enhanced Recovery After Surgery (ERAS) pathways integrate multimodal perioperative interventions which are designed to reduce physiological stress, facilitate early return of bodily function and reduce healthcare costs by reducing length of hospital stay (LOS) [1]. Much of the evidence for ERAS has been derived from patients undergoing lower gastrointestinal surgery. A meta-analysis of outcomes of patients undergoing major elective open colorectal surgery within an ERAS pathway, compared to conventional perioperative care, demonstrated ERAS pathways to be associated with significantly reduced postoperative complication rates (relative risk 0.53 [95 % confidence interval 0.41–0.69], *P* < 0.00001) and LOS (weighted mean difference −2.51 days [95 % CI, −3.54 to −1.47], *P* < 0.00001) [2]. There is, however, paucity of ERAS data originating from patients undergoing bariatric and metabolic surgery [3]. Indeed, the only published study from the United Kingdom was an observational case series of 406 laparoscopic Roux en-Y gastric bypass (LRYGB) patients that described a 'fast-track' anesthetic pathway [4]. However, the latter investigators did not utilize traditionally described ERAS interventions [1]. A recently reported randomized clinical trial examined an enhanced recovery pathway versus standard care following 78 patients undergoing laparoscopic sleeve gastrectomy [5]. In the latter study patients in the ERAS group demonstrated significantly shorter LOS and reduced hospital costs with no increase in occurrence of complications, although there was a 20 % re-admission rate in both ERAS and control groups [5]. Reasons for the delayed adoption of ERAS pathways within bariatric centres may include lack of robust evidence within this group of patients and concerns regarding the presence of complex high-risk medical co-morbidities that require specialist perioperative care. The aims of the present study were, therefore, to determine the feasibility and clinical outcomes following implementation of an Enhanced Recovery After Bariatric Surgery (ERABS) pathway in a high-volume tertiary referral bariatric centre.

## Materials and Methods

### Design and Setting

This prospective study collected data on consecutive patients undergoing primary bariatric procedures (LRYGB, sleeve gastrectomy and gastric banding) within an ERABS pathway. Procedures were performed over a 9-month period (15 April 2012 to 15 January 2013) at Imperial Weight Centre (London), a regional high-volume tertiary referral bariatric centre that employed four consultant bariatric surgeons and a bariatric fellow. The study had approval from the hospital regulatory authorities.

### Data Collection

Prospectively collected data included demographic details, baseline co-morbidities, operation performed and LOS. Data on occurrence of complications, mortality, re-admissions and re-operations were extracted retrospectively from medical case notes and emergency patient admission lists. The census date for data collection was 1 February 2013 (and included patients operated on until 15 January 2013). Data are presented as mean ± SD or median (interquartile range, IQR) as appropriate.

### Patient Enrolment

At the time of the study, regional National Health Service (NHS) commissioning criteria for bariatric surgery were followed. Surgery was indicated for patients with body mass index (BMI) ≥35 kg/m^2^ in the presence of obesity-related medical co-morbidities (defined as difficult to control hypertension, type 2 diabetes mellitus, obstructive sleep apnea or polycystic ovary syndrome). The direct surgical pathway utilized at Imperial Weight Centre included attendance at a patient seminar where information (oral and written) was delivered (by both a metabolic physician and bariatric surgeon) on the various medical and surgical treatments for morbid obesity. Thence followed attendance at a multi-disciplinary one-stop bariatric clinic where within a group setting, patients received presentations delivered by specialist obesity dieticians and clinical nurse specialists describing the perioperative pathway. They were also issued with the appropriate dietary advice sheets and incentive spirometers, ensuring they were familiarized with use of the latter. Pending surgical, medical and anesthetic reviews patients watched a Bariatric Surgery DVD to reiterate indications and expected outcomes from surgery. This ensured sufficient background knowledge was imparted prior to the medical and surgical reviews. Preoperative assessment was subsequently undertaken by a consultant bariatric anesthetist, a metabolic physician and a bariatric surgeon. Appropriate consultations with supporting specialties (renal, cardiac, respiratory and psychiatry physicians) were arranged as indicated. Patients with diabetes mellitus were reviewed by the metabolic physician and their medications adjusted to ensure optimized diabetes control preoperatively. All patients were listed for surgery once any necessary preoperative 'work-up' was completed and reviewed by the multi-disciplinary team. All patients undergoing primary surgery, who lived within a 2-h commute from the hospital (approximately 30 miles distance), and had appropriate home social support (an adult to care for them postoperatively) were included into the ERABS pathway. Otherwise, exclusion criteria included presence of renal (CKD III), cardiac or liver failure that necessitated postoperative admission to a high dependency unit or being wheelchair-dependent. Whilst most ERABS interventions were still utilized in excluded patients, postoperative analgesia, fluid requirements, mobilization and early discharge goals were tailored in a patient-specific manner.

### ERABS Interventions

**Fig. 1:**
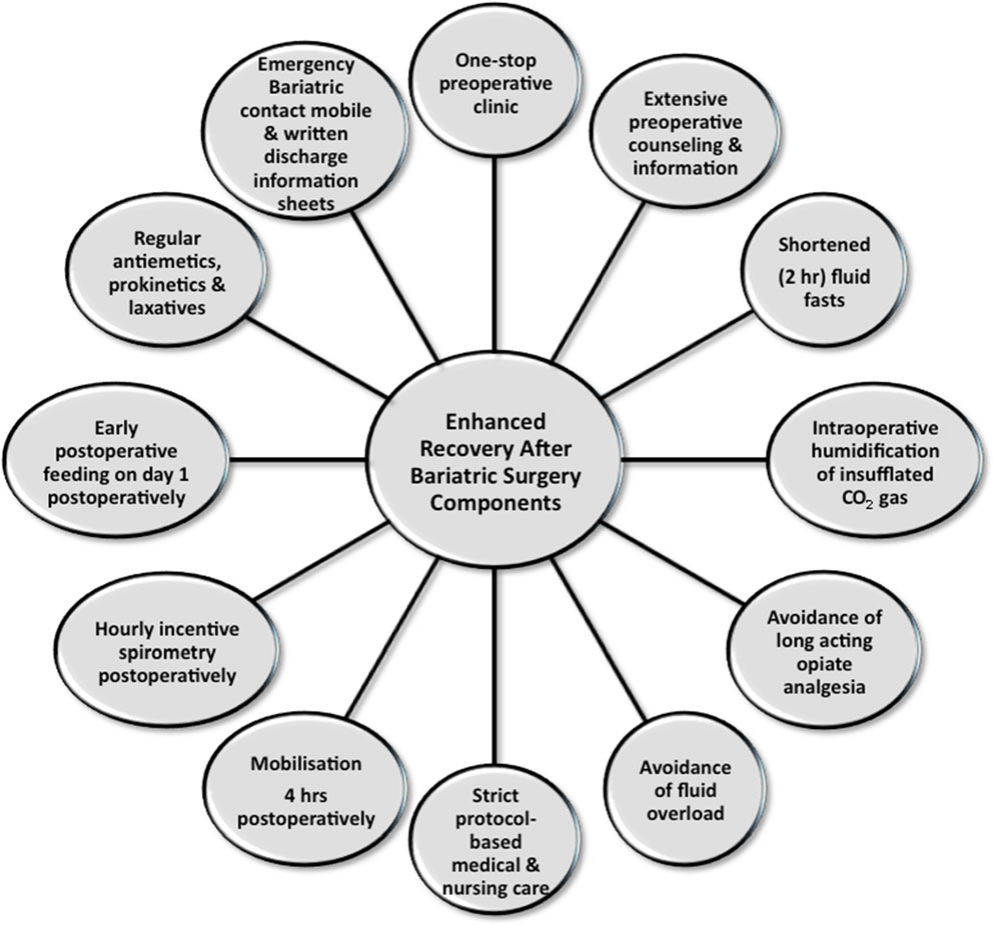
Perioperative Enhanced Recovery After Bariatric Surgery (ERABS) interventions used in this study

**Fig. 2:**
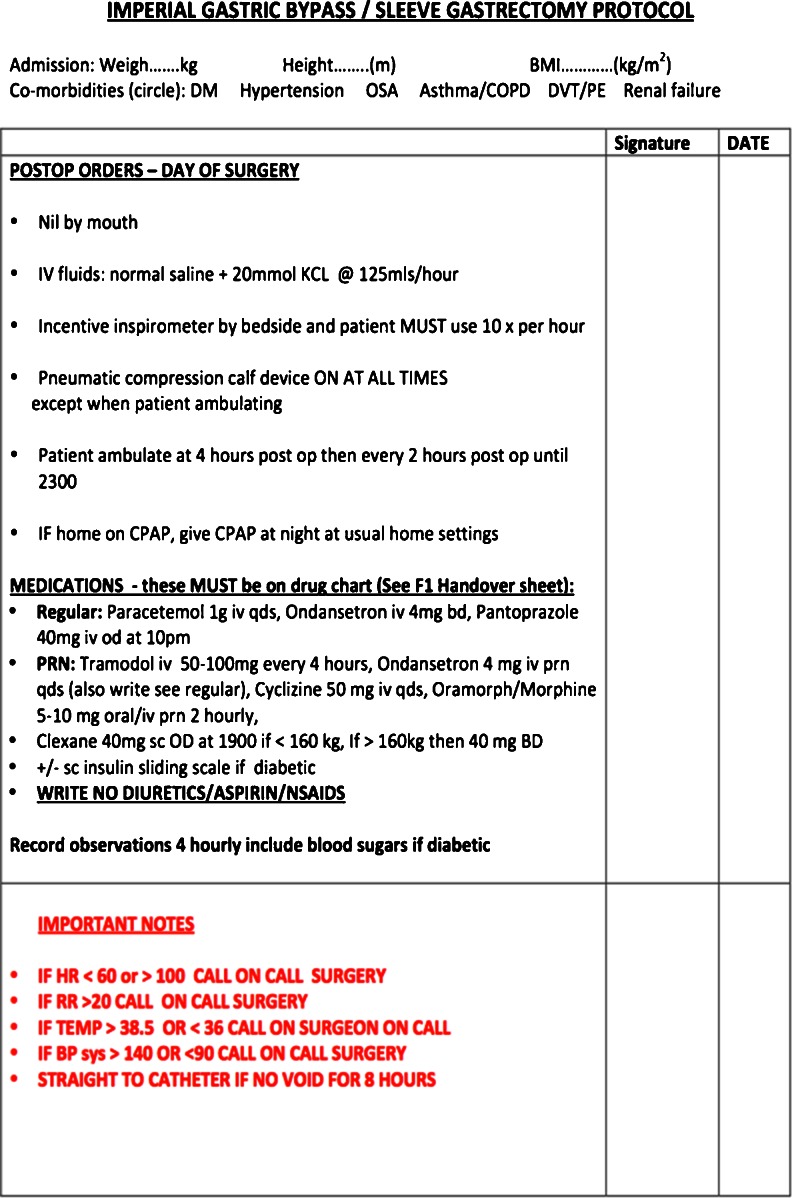
Postoperative medical and nursing protocol used following gastric bypass and sleeve gastrectomy

In accordance with the principles of multimodal ERAS pathways utilized in patients undergoing elective colorectal surgery [1], the ERABS pathway utilized at our bariatric centre (Fig. 1) included preoperative, intraoperative and postoperative interventions. *Preoperative* interventions included: extensive perioperative counselling, shortened fluid fasts (intake of clear liquids permitted up to 2 h pre-operatively) [6], and optimized operating schedules whereby patients were scheduled on operating lists on Mondays, Wednesdays and Fridays, based on their co-morbidities and anticipated postoperative specialist medical reviews and LOS. Patients with potential for day case discharge (e.g., gastric bands) were scheduled first on the operating list, whilst patients with complex co-morbidities were scheduled on Mondays and Wednesdays; anticipating specialist inpatient reviews which would be difficult to arrange during the weekend. As data on preoperative carbohydrate treatment were limited in the bariatric population [7] we did not utilize this intervention in this feasibility study. Patients undertook a preoperative low carbohydrate (800 kcal/day) liver-shrinking diet for a period of 2–4 weeks preoperatively (depending on preoperative BMI and sex). *Intraoperative* interventions included an optimized bariatric anesthesic protocol that included ramped head-up intubation and extubation, avoidance of long acting opioids (instead remifentanil was utilized intraoperatively), multimodal analgesia (use of intravenous Paracetamol, Diclofenac and Tramadol), volume controlled ventilation, use of high PEEP (6–8 cm/H_2_O) and permissive hypercapnea (end tidal CO_2_ >6.5 kPa) the latter used for its vasodilatory effects (to allow intra-abdominal bleeding to be detected intraoperatively). Furthermore, no steroids or benzodiazepines were utilized intraoperatively, and the BIS EEG VISTA™ Monitor System (Aspect Medical Systems, Massachusetts, USA) was used to guide and minimize intraoperative anesthetic requirements (BIS to less than 60). Patients typically received 1.5–2 l of crystalloid infusion intraoperatively and local anesthetic was infiltrated at surgical port sites at the start and end of surgery (typically, a total of 40 ml 0.25 % Bupivacaine with 1:200,000 Adrenalin was used). Intraoperative humidification and heating of insufflated CO_2_ gas using the HumiGard™ system (Fisher & Paykel Healthcare Ltd, Auckland, New Zealand) was also used as in our experience of this intervention, patients developed less postoperative pain and use of humidified warmed CO_2_ reduced laparoscopic lens fogging enhancing speed and clinical efficiency. Alongside the WHO checklist, a dedicated bariatric patient 'time-out' was undertaken identifying patient-specific co-morbidities to the theatre team. Patients were positioned at 60° reverse Trendelenburg to ensure optimal intraabdominal space was available in the supracolic compartment. Laparoscopic RYGB were performed in an antecolic–antegastric fashion (typically 50 cm biliopancreatic limb, 100 cm Roux limb length) using an end-to-side (but functional end-to-end) linear stapled gastrojejunal anastomotic technique. Sleeve gastrectomies were sized using a 34F orogastric tube and utilizing Seamguard® (GORE®) staple line reinforcement. The *pars flaccida* approach was used to place gastric bands and a gastro-gastric tunnel was formed in all cases to reduce the incidence of band slippage. Nasogastric tubes and urinary catheters were not routinely used and use of surgical drains was reserved for patients whom the surgeon considered at increased postoperative risk of bleeding. *Postoperative* interventions included protocol-based care using a didactic protocol (Fig. 2) that ensured full mobilization within 4 h of the end of surgery, hourly use of incentive spirometry, a postoperative intravenous fluid balance regimen that avoided fluid overload (previously shown to delay return of gut function [8]) and the administration of regular multimodal (opiate sparing) analgesia (intravenous Paracetamol 1 g QDS, Tramadol melts 50–100 mg QDS), antiemetics (Ondansetron 4 mg TDS), prokinetics (Metoclopromide 10 mg TDS), and laxatives (Lactulose 15 ml BD). Compliance with the aforementioned post-operative interventions was ensured by asking nursing staff to sign opposite each intervention used. Although not formally assessed, lack of compliance with these interventions occurred in only a few patients when certain devices/drugs were out of stock on the surgical ward. A subcutaneous insulin sliding scale regimen was used postoperatively for type 2 diabetic patients with good effect and avoided problems with maintaining 'insulin-exclusive' additional intravenous access postoperatively in these patients. Patients were reviewed postoperatively three times daily by the bariatric fellow at 7:30 am, 1 pm and 4:30 pm to ensure appropriate clinical and discharge concerns were addressed early. Liquid diet was commenced on the first postoperative day without need for routine contrast studies to confirm anastomotic integrity. The latter, however, were selectively used in patients in whom an unsatisfactory intra-operative air-leak test was obtained or when clinically indicated postoperatively. Written discharge information sheets were issued to patients on the morning ward round on the first postoperative day to ensure sufficient time to read and understand information contained therein. Information on the postoperative recovery, diet, alarm symptoms, and a dedicated 24-h emergency bariatric mobile number were also issued to patients. Discharge criteria included the presence of all of: (1) able to drink 1.5 l of fluid per day and tolerating liquid diet; (2) pain adequately controlled with oral analgesia; (3) adequate mobilization; (4) presence of a supervising adult at home; and (5) understand and accept the written information sheets provided. Early postoperative clinical review (proforma-guided) on day 10 was undertaken by the bariatric clinical nurse specialists who had access to an immediate bariatric surgical consult if necessary; with the aim of eliciting early postoperative concerns and to reiterate postoperative dietary instructions to patients.

## Results

During the 9-month study period a total of 280 bariatric procedures were performed. Of these 226 (80.7 %) primary bariatric procedures were undertaken and included in the present study. The remaining 54 patients underwent revisional surgery or met the aforementioned exclusion criteria. There were 150 (66 %) LRYGB, 47 (21 %) sleeve gastrectomies and 29 (13 %) gastric bands. No patients enrolled within the ERABS pathway were subsequently excluded operatively or postoperatively. Baseline demographic and co-morbidity data are shown in Table 1. All procedures were successfully completed laparoscopically. No anastomotic or staple line leaks/bleeds were encountered in this series. Postoperative morbidity and LOS are given in Table 2. One postoperative death occurred at 36 h postoperatively. The latter resulted from massive pulmonary embolus that occurred in a high-risk patient (previous history of pulmonary embolus) despite insertion of preoperative IVC filter and postoperative anticoagulation with low molecular weight heparin and use of intermittent pneumatic compression device. One diagnostic re-laparoscopy at 48 h was performed for suspected intra-abdominal sepsis following sleeve gastrectomy and extensive adhesiolysis, however, no abnormalities were found at surgery (30-day re-operation rate 0.4 %). Thirty-day hospital re-admissions occurred in six (2.7 %) patients due to development of non-specific abdominal pain (all investigations negative, three patients), constipation (one patient), diarrhea and dehydration due to lactose intolerance that developed 3 weeks post LRYGB (one patient) and extensive lower limb deep venous thrombosis that followed an unsuccessful attempt at removing a temporary IVC filter (one patient).

**Table 1:** Baseline demographic and co-morbidity data for the 226 participants of this study

Age, mean ± SD (years)	45 ± 11
Sex (%)	163 female (72 %)
Overall BMI, median (IQR) kg/m^2^	44.9 (41.0–49.0)
▪ Gastric bypass patients	44.7 (41.6–48.0)
▪ Sleeve gastrectomy patients	45.0 (41.2–50.7)
▪ Gastric band patients	43.0 (38.8–49.9)
Medical co-morbidity, number (%)	
▪ Hypertension	90 (40 %)
▪ Type 2 diabetes mellitus	77 (34 %)
▪ Obstructive sleep apnea	54 (24 %)
▪ Limited mobility^a^	20 (9 %)

*IQR* interquartile range

^a^Limited mobility patients used walking sticks or frames to mobilize due to arthritis or back pain. Wheelchair-bound patients were excluded from enrolment into the Enhanced Recovery pathway

**Table 2:** Postoperative morbidity and length of hospital stay

Postoperative morbidity, number of patients (%)	10 (4.4 %)
▪ Respiratory morbidity (5 LRTI, 1 type II respiratory failure)	6
▪ Thromboembolic complications (one DVT, one PE)	2
▪ Post-operative bleed (dropped Hb, managed conservatively)	1
▪ Postoperative morbidity, number of patients (%)	1
Length of hospital stay, mean ± SD (days)	
▪ Gastric band	0.69 ± 0.81
▪ Gastric bypass	1.88 ± 1.12
▪ Sleeve gastrectomy	2.30 ± 1.69
Day-case discharge successful for gastric band patients	14 (48 %)
Discharge successful on first postoperative day, number (%)	
▪ Gastric bypass	56 (37 %)
▪ Sleeve gastrectomy	13 (28 %)

Presence of postoperative LRTI requiring antibiotic treatment was determined either clinically (newly developed cough productive of green/brown phlegm with signs of sepsis) or radiologically (presence of pulmonary opacities/infiltrates coupled with clinical signs in keeping with LRTI)

*DVT* lower limb deep venous thrombosis, *Hb* hemoglobin, *LRTI* lower respiratory tract infection, *PE* pulmonary embolus

## Discussion

This prospective study has demonstrated that implementing an ERABS pathway was feasible, safe and associated with low 30-day complication (4 %) and hospital re-admission (2.7 %) rates. Our pragmatic study design set out to determine whether implementation of an ERABS pathway in a 'real-life' setting would lead to outcomes equivalent to those seen within a research setting [5]. As such we deliberately limited the exclusion criteria to ensure most patients encountered in clinic would be eligible for inclusion. Similarly, patients operated on Fridays were not excluded from the series despite the likelihood that their discharge may have been delayed whilst cared for by the weekend emergency surgical teams (as opposed to usual bariatric team).

At the time of this study, local NHS commissioning and eligibility criteria for bariatric surgery required presence of BMI ≥ 35 kg/m^2^ along with presence of an obesity-related co-morbidities. Thus the majority of patients in this series had a combination of poorly controlled hypertension, diabetes mellitus and obstructive sleep apnea. Almost 10 % of the series had poor mobility, a factor that may have hampered postoperative mobility, recovery and resulted in delayed discharge. That said, mean LOS was 1.88 and 2.30 days for LRYGB and sleeve gastrectomy patients, respectively. Furthermore, almost a third of gastric bypass and sleeve gastrectomy patients were successfully discharged on the first postoperative day; and half of gastric band patients were performed within a day-case setting. Importantly, these results were achieved with an impressive 2.7 % 30-day hospital re-admission rate, which compares to the 20 % readmission rate encountered in a recently reported randomized study of sleeve gastrectomy patients within an ERAS pathway [5]. Whilst it was theoretically possible that patients may have presented postoperatively to other hospitals this was unlikely as our patients were strongly counselled to contact the emergency bariatric mobile (call logs were kept) and re-present to our emergency department in the event of any problems. Furthermore, postoperative outpatient clinic review of these patients did not elicit attendance at other hospitals.

We used traditional ERAS components within our pathway accepting there was a relative lack of evidence base within the bariatric population [3]. However, several of the components included in our protocol had a strong evidence base from lower gastrointestinal surgery [1] and as such are now widely considered best surgical practice. Specific components of traditional ERAS protocols [1] that were not included in our protocol included preoperative carbohydrate treatment which has been demonstrated in a recent meta-analysis to be associated with decreased LOS following major elective abdominal surgery [7]. However, there are presently no studies examining the gastric emptying and the metabolic effects of these preconditioning drinks [9] within the bariatric population; of whom up to a third (as in this series) may have diabetes mellitus.

Standardization of anesthetic protocols, surgical technique, and use of a didactic medical and nursing postoperative protocol may have contributed to the good clinical outcomes (low complication and re-admission rates) encountered in this series. The later was developed from experience gained at our bariatric centre over the past 7 years which included over 2,000 gastric bypasses and 500 sleeve gastrectomies. Of equal importance was the unit policy to ensure patients were recovered postoperatively in dedicated bariatric beds that were manned by bariatric-trained nursing staff. In keeping with this, there is evidence that standardization of perioperative care may be associated with improved outcomes [10]. Whilst significant variation exists within bariatric practice in the United Kingdom [11], of more importance is the standardization of unit policy to ensuring adequate induction and familiarity of staff with protocols used therein. This study has a number of limitations. Firstly, it lacks a control group with which to compare outcomes from the ERABS pathway. Similarly, we did not present data on LOS prior to implementation of the ERABS protocol, as the former were not available. Secondly, postoperative LOS is used as a surrogate marker of recovery. Thirdly, we did not seek to perform an economic analysis of the effects of ERABS, however, this data has recently been reported from a randomized trial [5].

## Conclusion

This study has demonstrated that applying an ERABS protocol was feasible, safe, associated with low morbidity, acceptable LOS and low 30-day hospital re-admission rates. The presence of multiple medical co-morbidities should not preclude use of such a protocol within bariatric patients.
